# A Bayesian Approach to Account for Misclassification and Overdispersion in Count Data

**DOI:** 10.3390/ijerph120910648

**Published:** 2015-08-28

**Authors:** Wenqi Wu, James Stamey, David Kahle

**Affiliations:** Department of Statistical Science, Baylor University, One Bear Place #97140, Waco, TX, 76706, USA; E-Mails: wenqi_wu@baylor.edu (W.W.); james_stamey@baylor.edu (J.S.)

**Keywords:** misclassification, count data, overdispersion

## Abstract

Count data are subject to considerable sources of what is often referred to as non-sampling error. Errors such as misclassification, measurement error and unmeasured confounding can lead to substantially biased estimators. It is strongly recommended that epidemiologists not only acknowledge these sorts of errors in data, but incorporate sensitivity analyses into part of the total data analysis. We extend previous work on Poisson regression models that allow for misclassification by thoroughly discussing the basis for the models and allowing for extra-Poisson variability in the form of random effects. Via simulation we show the improvements in inference that are brought about by accounting for both the misclassification and the overdispersion.

## 1. Introduction

Epidemiologic studies often have data that are subject to a wide array of different types of error. Measurement error, unmeasured confounding, and selection bias are all examples of sources of biased estimators and reduced power for hypothesis tests [1,2]. For continuous covariates, the problem of imperfect assessment is referred to as measurement error. A thorough review of measurement error, including remedial measures, can be found in Carroll *et al.* [3]. When considering discrete covariates, binary exposure variables are often measured with error, and such error is known to yield biased estimators [4,5]. Misclassification in ordinal covariates has also been considered [6]. In this binary setting, misclassification is communicated in the language of diagnostic tests: sensitivities and specificities. On the response side of the model, imperfect assessment for both binary/categorical (“response misclassification”) and count variables has also been considered [7,8].

In the case of Poisson regression, two approaches have been taken to correct for misclassification error, one frequentist and one Bayesian. From the frequentist perspective, Edwards *et al.* [1] consider the problem using maximum likelihood (ML) techniques assuming fixed and known sensitivity and specificity. From the Bayesian perspective, Stamey *et al.* [9] are able to free the sensitivity and specificity from being fixed or known by making use of validation data or informative priors. Both approaches have strengths and weaknesses. Treating the sensitivity and specificity as known, as done by Edwards *et al.* [1], is a strong assumption when these values are determined from small prior data sets or expert opinion. However, the “fixed” sensitivity and specificity approach is usually enhanced by performing a sensitivity study where a range of values for the sensitivity and specificity are plugged in and the impact to the estimated parameters of interest is noted. On the other hand, the Bayesian model of Stamey *et al.* [9], which fully accounts for the uncertainty in estimation, may require considerable computational time for the Markov chain Monte Carlo (MCMC) to converge and might also require a sensitivity analysis. Assuming these parameters are fixed and known reintroduces identifiability into the model so that the parameters are estimable from either the ML or Bayesian perspective.

In some cases, extra-variability beyond what is allowed for with a Poisson distribution is observed in the data, an effect called overdispersion. Gorman *et al.* [10] consider the effect of non-response on estimation of alcohol related outcomes using a Poisson model with overdispersion. Milner *et al.* [11] consider this problem in an analysis of the impact of the 2007 recession on suicide rates in Australia. Paulino *et al.* [12] consider a misclassified binomial model with overdispersion and allow for extra variability in their model with a random effect. In this paper we focus on the case where sampling is done either in clusters or longitudinally, motivating the need for a random effects model.

Statisticians have done considerable research into developing methods for correcting observational data for biases due to misclassification, measurement error, unmeasured confounding, *etc*. In this paper we focus on the important case of count data with misclassification and provide a cohesive estimating procedure for inference for a range of models of interest, specifically fixed and random effects models and the cases of known and unknown misclassification rates. Our goal is to demonstrate and show the value and of methods for accounting for bias in epidemiologic models with the Bayesian approach. Our paper is organized as follows. In Section 2 we overview the Poisson model with misclassification for both fixed and random effects. In Section 3 we discuss the prior distributions used for all parameters in the models and methods used for posterior inference. In Section 4 we consider the analysis of a single synthetic dataset. We discuss the results of simulation studies in Section 5 and give some concluding comments in Section 6.

## 2. The Model

In this section we introduce the model of interest using an example from Edwards *et al.* [13]. To aid in the description, we build the model with increasing levels of complexity communicated through the language of directed graphical models, also called Bayesian networks. In that language, fixed known quantities are represented as dots and variables are represented as nodes (circles), which are shaded if they are to be observed as data. The directed edges (arrows) represent dependence relationships, with the defining property being that a node is conditionally independent of any node that is not one of its descendants given its parents. Nodes that have a double lining are considered deterministic/known given their parents. For more background on graphical model, we find Koller and Friedman [14] to be an excellent, extensive treatment.

**Figure 1:**
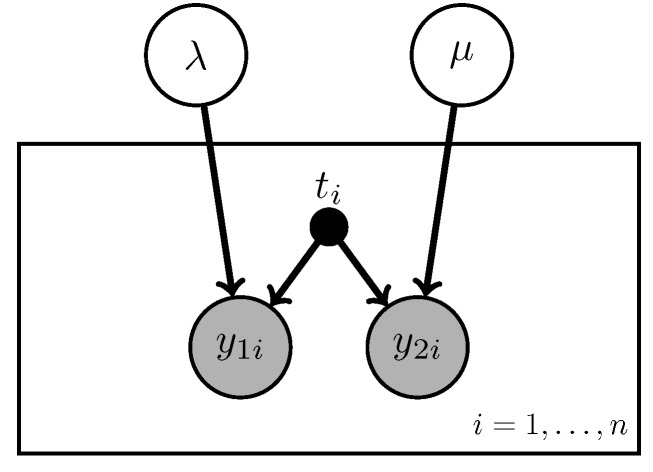
The naive baseline model: the number of deaths due to cancer ($y_{1i}$) and non-cancer ($y_{2i}$) follow a Poisson distribution with constant parameters.

The overarching question of interest in this article is to understand the rate at which individuals succumb to lung cancer and later, determinants of that rate. To begin modeling death due to lung cancer or alternative causes we temporarily overlook covariates. The data are based on death certificate information, which consists of the number of deaths due to lung cancer ($y_{1i}$) and those due to other causes ($y_{2i}$), here the index *i* runs from 1 to *n* and represents a number of observations, e.g., counties where death certificate information is obtained. Both of these are counts gathered over the same opportunity size $t_{i}$. In these scenarios it is standard practice to assume that each of these counts follow a Poisson distribution with rate parameters *λ* and μ, respectively. The graphical model corresponding to this situation is presented in Figure 1. Note that the rectangle (“plate notation”) is graphical shorthand for iterated relationships, so that the same *λ* and μ are are the rates in each location. This is a naive assumption that will be relaxed shortly.

To make the model more realistic, we let the rates vary depending on various covariates. In particular, we assume the rates are related to the covariates through log-linear models:

(1) $$\log\left(\lambda_{i}\right)\;\;=\;\;\beta_{0}+\beta_{1}X_{i}+\sum_{j=1}^{p}\beta_{j}Z_{ij}\;\;=\;\;\beta_{0}+\beta_{1}X_{i}+Z_{i}^{\prime }\beta$$

and

(2) $$\log\left(\mu_{i}\right)\;\;=\;\;\gamma_{0}+\gamma_{1}X_{i}+\sum_{j=1}^{p}\gamma_{j}Z_{ij}\;\;=\;\;\gamma_{0}+\gamma_{1}X_{i}+Z_{i}^{\prime }\gamma$$

**Figure 2:**
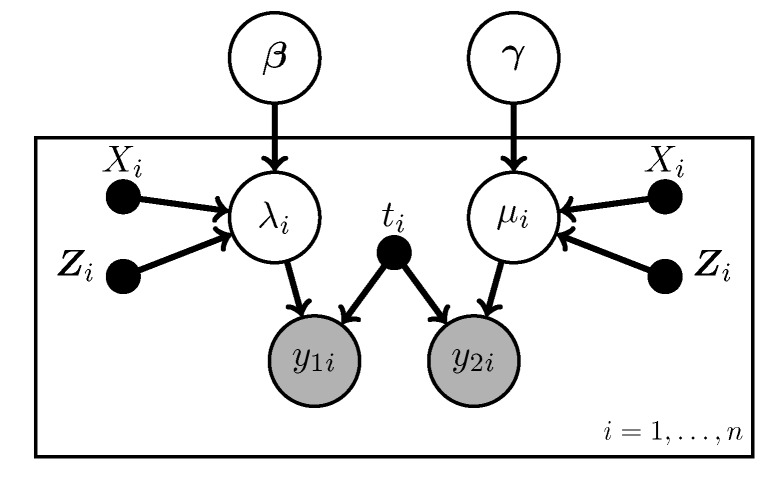
The no-misclassification Poisson regression model.

Here $X_{i}$ is the main exposure of interest for observation *i* while the $Z_{ij}$ are other covariates associated with it; *p* is the number of non-treatment covariates. This model is diagramed in Figure 2. Note that bolded symbols represent vector quantities.

Outcomes such as lung cancer deaths are based on death certificate information, which is well known to be subject to potential misclassification [7,15]. In particular, we note that it may be naive to consider the observed quantities to be the true cancer and non-cancer death counts, as indicated in the two previous models. Instead, we add an additional layer of complexity to the model by considering the observed data to be the counts of deaths that are *labeled* as due to lung cancer ($w_{1i}$) or other causes ($w_{2i}$). The true number of deaths due to lung cancer ($y_{1i}$) and non-lung cancer ($y_{2i}$) are thus considered unobserved.

**Figure 3:**
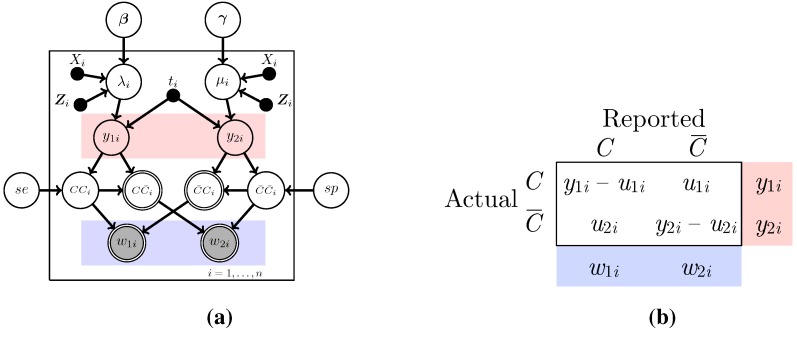
The Poisson regression model with misclassification; (**a**) The graphical model representation of the model. se denotes the sensitivity of the classifier, and sp denotes its specificity; (**b**) The contingency table representation of the data. $y_{1i}$ ($y_{2i}$) is the true number of deaths due to lung cancer (non-lung cancer). $u_{1i}$ ($u_{2i}$) is the number of true number of lung cancer (non-lung cancer) deaths misclassified. $w_{1i}$ ($w_{2i}$) is the observed number of deaths due to lung cancer (non-lung cancer). Note that *C* and $\bar{C}$ denote correctly classified and misclassified, respectively.

Since $y_{1i}$ and $y_{2i}$ are not observed, the error prone data $w_{1i}$ and $w_{2i}$ are used in the analysis instead. The misclassified counts $w_{1i}$ and $w_{2i}$ depend on the true counts $y_{1i}$ and $y_{2i}$ and misclassified counts $u_{1i}$ and $u_{2i}$ (also unobserved) in the following way. The count $u_{1i}$ is the number of cancer deaths incorrectly labeled as non-cancer and $u_{2i}$ is the number of non-cancer deaths incorrectly labeled as due to cancer. We then have $w_{1i}=y_{1i}-u_{1i}+u_{2i}$ and $w_{2i}=y_{2i}+u_{1i}-u_{2i}$. The relationships between the observed $w_{1i}$ and $w_{2i}$ and the unobserved variables are shown in diagram and table form in Figure 3. Note that $CC_{i}$ represents the true cancer deaths (correct) classified as cancer deaths; $C{\bar{C}}_{i}$ the true cancer deaths (incorrectly) classified as non-cancer deaths; and similarly for $\bar{C}C_{i}$ and $\bar{C}{\bar{C}}_{i}$.

A simple derivation reveals that, like the unobserved counts, the observed counts also follow Poisson distributions, but the rates are functions of all the parameters. The likelihood for the observed data is

(3) $$p\left(w_{1i},w_{2i}\right)\;\propto \;{\left(\lambda_{i}se+\mu_{i}\left(1-sp\right)\right)}^{w_{1i}}{\left(\lambda_{i}\left(1-se\right)+\mu_{i}sp\right)}^{w_{2i}}\exp\left\{-\left(\lambda_{i}+\mu_{i}\right)n_{i}\right\}$$

where se is the probability a lung cancer death is correctly labeled as lung cancer; sp is the probability a death due to all other causes are correctly labeled as not being due to lung cancer; and $\lambda_{i}$ and $\mu_{i}$ are the covariate specific death rates for the *i*th observational unit. Thus the $w_{1i}$ and $w_{2i}$ are biased for the rates $\lambda_{i}$ and $\mu_{i}$. For instance, $w_{1i}$ provides information only about the quantity $\lambda_{i}se+\mu_{i}\left(1-sp\right)$, and without additional information, there is no way to disentangle a direct estimate for $\lambda_{i}$. In other words, accounting for misclassification with the two parameters of sensitivity and specificity overparameterizes the model in a way that demands to be addressed. Edwards *et al.* [13] consider se and sp known and provide a method to obtain the maximum likelihood estimators (MLEs) while Stamey *et al.* [9] assume information about se and sp exist not as point estimates but rather in the form of probability distributions with which one can perform a Bayesian analysis, *i.e*., prior distributions. Here, we investigate both the fixed and unknown approaches via the Bayesian paradigm.

The model described above is the same one used in both Stamey *et al.* [9] and Edwards *et al.* [13] and does not allow for extra Poisson variability. If the sampling is done in clusters, or if there are repeated measures, a random effects model may be appropriate as an alternative to the fixed effects model already described. When using random effects, the models listed in the random effects case the log-linear models in Equation (1) and Equation (2) become

(4) $$\log\left(\lambda_{i}\right)\;\;=\;\;\beta_{0}+\beta_{1}X_{i}+\sum_{j=1}^{p}\beta_{j}Z_{ij}+e_{\lambda_{k[i]}}\;\;=\;\;\beta_{0}+\beta_{1}X_{i}+Z_{i}^{\prime }\beta +e_{\lambda_{k[i]}}$$

and

(5) $$\log\left(\mu_{i}\right)\;\;=\;\;\gamma_{0}+\gamma_{1}X_{i}+\sum_{j=1}^{p}\gamma_{j}Z_{ij}+e_{\mu_{k[i]}}\;\;=\;\;\gamma_{0}+\gamma_{1}X_{i}+Z_{i}^{\prime }\gamma +e_{\mu_{k[i]}}$$

Here, $e_{\lambda_{k[i]}}∼N\left(0,\sigma^{2}\right)$ and $e_{\mu_{k[i]}}∼N\left(0,\sigma^{2}\right)$. This model is sometimes referred to as a random intercept model. If a more complicated hierarchical structure is desired, the slopes could also be modeled, but that is beyond what we are interested in this work.

We initially assume the random effects come from a common distribution to limit the number of parameters required to be estimated and in general, these variances would unlikely be largely different. However, this is a very strong assumption so we also provide a more flexible model. For this more general model, we assume the $e_{\lambda_{k[i]}}$ and $e_{\mu_{k[i]}}$ are bivariate normal

(6) $$e∼N_{2}\left(\left[\begin{array}{c} 0\\ 0 \end{array}\right],\left[\begin{array}{cc} \sigma_{1}^{2} & \rho \sigma_{1}\sigma_{2}\\ \rho \sigma_{1}\sigma_{2} & \sigma_{2}^{2} \end{array}\right]\right)$$

## 3. Priors and Posterior Inference

**Figure 4:**
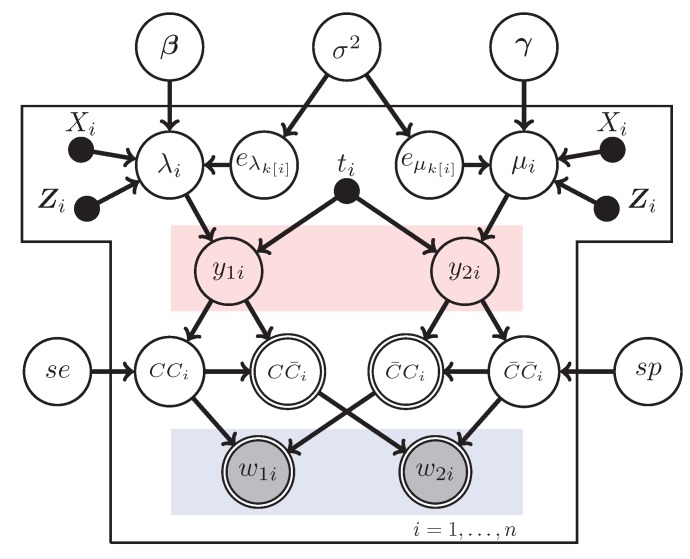
The random-intercept Poisson regression model with misclassification.

We consider four different models. Model 1 is a Bayesian version of the model in Edwards *et al.* [13]. That is, it is the fixed effects Poisson model and the diagnostic parameters se and sp are assumed to be known, as in Figure 3 with se and sp fixed. Model 2 is the model of Stamey *et al.* [9] which is also a fixed effects Poisson model but allows for uncertainty in se and sp by replacing the fixed values with beta priors for those two parameters; this is Figure 3. Model 3 extends Model 1 by adding a random effect to account for clustered sampling designs, which is Figure 4 with fixed se and sp , and Model 4 adds a random effect to Model 2, which is Figure 4.

For all models we assume relatively diffuse independent normal priors for the regression coefficients. Specifically, we have

(7) $$\beta_{j}∼N\left(0,10\right),\;j=0,1,...,p$$

and

(8) $$\gamma_{j}∼N\left(0,10\right),\;j=0,1,...,p$$

For all the simulations we consider, a prior standard deviation of 10 for the regression parameters leads to a very diffuse prior relative to the likelihood. In practice, the user should consider likely values for these parameters when choosing the standard deviation for these priors. For a Poisson regression, priors allowing for values from –20 to 20 would almost always be sufficient; on the scale of the Poisson rate this would allow for multiplicative effects ranging from 0 to about 500 million.

For the diagnostic parameters se and sp, for models 2 and 4 where they are considered unknown, we assume independent beta priors,

(9) $$se∼\text{Beta}\left(a_{se},b_{se}\right)$$

and

(10) $$sp∼\text{Beta}\left(a_{sp},b_{sp}\right)$$

The beta is a flexible distribution allowing for a wide variety of shapes. Also, it is the conjugate prior for a binomial likelihood, so if validation data for both the true and fallible results are available, the values can be used to specify the *a*’s and *b*’s. For instance, Sposto *et al.* [7] consider the impact of misclassification on cancer rate estimates in Hiroshima and Nagasaki. Most of their data is based on fallible death certificate information, but for a subset of the deaths autopsies were performed that could be treated as a “gold standard” (*i.e*., infallible or perfectly classified data). Suppose for instance that in the validation data there are *m* autopsied subjects known to have died from cancer but the death certificates only correctly labeled *w* of them. The likelihood for this data is thus binomial:

(11) mwsew(1-se)m-w

This data leads directly to a $\text{Beta}\left(w+1,m-w+1\right)$ prior for the sensitivity, se. Combining the autopsy and death certificate information the same sort of prior for sp can be obtained. Alternatively, the beta priors can be viewed as a mechanism to perform a sensitivity analysis. While Edwards *et al.* [13] assume the se and sp are known, they consider a wide range of values as part of a sensitivity analysis. Using similar logic, the prior parameters for se and sp can be selected so that the priors reflect the range for the sensitivity and specificity of interest. There are free packages such as Chun-Lung Su’s Beta-Buster, implemented in R’s **epiR** package [16], that aid in the specification of such beta priors.

The random effects standard deviation in Models 3 and 4 is the final parameter requiring a prior distribution. The conjugate prior for a variance in this situation is the inverse gamma, so a Inv-Gamma $\left(0.001,0.001\right)$ is often used. Gelman [17] find that both a half-Cauchy distribution and a uniform distribution perform better as priors than the inverse gamma. Here, we use a Unif(0,D) prior where *D* is an upper value for the support that is chosen to minimize influence on the posterior. A value of 5 will often be large enough, but should be checked in each unique situation.

If the correlated random effects model is used instead of the equal variance model, then priors for $\sigma_{1}$, $\sigma_{2}$, and ρ are required. In the absence of substantial prior information, uniform priors for all three parameters would often be used. Specifically, $\sigma_{1}∼\text{Unif}\left(0,B_{1}\right)$, $\sigma_{2}∼\text{Unif}\left(0,B_{2}\right)$ and ρ∼Unif(-1,1).

We fit the models using Markov chain Monte Carlo (MCMC) methods via the free package OpenBUGS. The OpenBUGS code used for fitting the models and the R code used to generate all the data for simulations is available upon request. As always, when using MCMC methods care must be taken to ensure the validity of the results. For the models where the misclassification parameters are assumed unknown, there were times when the chains did not mix sufficiently well, indicating a lack of convergence clearly visible in the trace plots of the MCMC. This is not unusual for overparameterized models such as these. When chains illustrate a lack of convergence, remedial measures including increasing the number of burn-in iterations and thinning the chains improved convergence. Another important issue is starting values for the chains. The test parameters, se and sp must sum to be greater than 1 or the classifying technique is actually worse than random guessing. Thus starting values for se and sp should be chosen so that se+sp>1.

## 4. Simulated Example

We consider a simulated example to illustrate the new random effects model and how the models can be used for sensitivity analysis. We imagine a scenario where interest is in the relationship between lung cancer deaths due to a particular exposure. We suppose the data are clustered with each cluster containing four observations. Thus the random effects model is appropriate. We generated the data from using Equations (4) and (5) with three binary covariates, with a total sample size of N=32 observations and K=8 clusters. The parameter values chosen resulted in models

(12) $$\log\left(\lambda_{i}\right)=-1+0.5x_{1ij}-0.5x_{2ij}+0.1x_{ij}+e_{\lambda_{k[i]}}$$

(13) $$\log\left(\mu_{i}\right)=-2-0.3x_{1ij}+0.2x_{2ij}+0.5x_{ij}+e_{\mu_{k[i]}}$$

To generate the data, instead of assuming a common standard deviation for the random effects, we assumed $\left(e_{\lambda_{k[i]}},e_{\mu_{k[i]}}\right)$ come from a bivariate normal distribution with means of 0, $\sigma_{1}=0.2$, $\sigma_{2}=0.4$, and ρ=0.5. We assumed 1000 person-months for each observation. Finally, we assume the true sensitivity is 0.75 and true specificity is 0.8. The counts for each observation ranged from a low of 148 to a high of 613, so this would be a relatively large observational study.

Suppose an expert thinks the most likely value for the sensitivity is 0.7 and it could (5% chance) be as low as 0.6 and as high as 0.8. For the specificity, the most likely value is 0.8 with a 5% chance of being lower than 0.7 and as high as 0.9. These beliefs can be roughly summarized into $se∼\text{Beta}\left(35,15\right)$ and $sp∼\text{Beta}\left(40,10\right)$.

Before discussing the overall results and illustrating how to use the methods to perform a sensitivity analysis, we compare estimates for the correlated random effects and single standard deviation model. Even though $\sigma_{2}$ is twice $\sigma_{1}$, the posterior estimates for the regression parameters are almost identical. For instance, the primary parameter of interest is $\beta_{1}$. The posterior mean and 95% interval are 0.541 and (0.454, 0.646) for the correlated model and 0.540 and (0.452, 0.643) for the single variance model. Due to the over-parameterization that is already in the model due to the misclassification, unless strong evidence against the equal standard deviation model exists, we recommend using it instead of the correlated random effects model.

**Figure 5:**
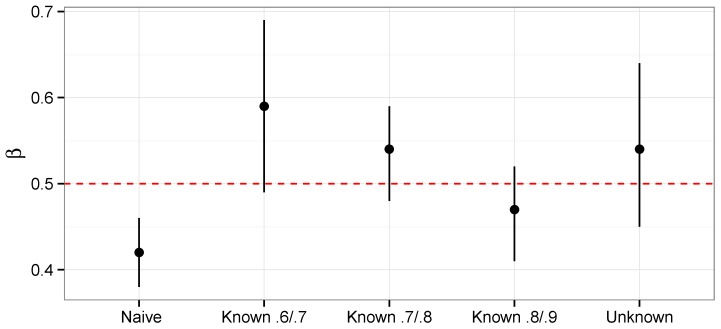
Posterior means and 95% credible sets for sensitivity analysis of $\beta_{1}$ (with true value 0.5).

One advantage of using informative priors on se and sp instead of using fixed values is the analysis of the data doubles as a sensitivity analysis. There is essentially no information in the data on se and sp. Thus the posterior distributions are approximately the same as the prior distributions. If it is desired to assure that the priors and posteriors for the misclassification parameters completely match, the cut function in WinBUGS and OpenBUGS can be used so that the priors for se and sp exactly match the posteriors. In this case, the Bayesian analysis is a version of Monte Carlo sensitivity advocated for in Steenland and Greenland [18]. As mentioned before, $\beta_{1}$ is the primary parameter of interest. In Figure 5 we plot the posterior mean and 95% interval for $\beta_{1}$ for the naive model where the misclassification is ignored, Model 4, where se and sp are given prior distributions and several versions of Model 3. For Model 3, where se and sp are fixed, we consider the following (se,sp) pairs: (0.8, 0.9), (0.7, 0.8) and (0.6, 0.7). These pairs represent the most optimistic, most likely, and most pessimistic values according to the expert. The misclassification in the data biases the estimates towards the null value of 0, which is why the naive model has the lowest posterior mean. Note also that the interval for the naive model does not contain the true value. For Model 3, the pessimistic choice of (0.6, 0.7) shifts $\beta_{1}$ upwards the most. One approach to the sensitivity analysis would be to take the upper limit of the interval for the (0.6, 0.7) posterior and the lower limit of the (0.8, 0.9) posterior. This would yield an interval of (0.41, 0.69). Another option would be to simply use the interval that corresponds to the most likely pair of (0.7, 0.8). This would yield an interval of (0.48, 0.59). What is interesting is that the Monte Carlo sensitivity analysis of Model 4 with a 95% interval of (0.45, 0.64) provides a very nice intermediate step between these two extremes. In the next Section, we investigate the operating characteristics of these procedures via simulation.

## 5. Simulation Study

We conducted a series of simulations to illustrate the performance of the four models under various situations. For these simulations we focused on inference, specifically the average of the posterior means, along with width and coverage of 95% intervals. The code we use is easily modified to include hypothesis testing and monitor quantities such as Type I error rates and power. For the simulation we assumed three binary covariates and for each covariate pattern we assumed the person-time, $t_{i}=1000$. In anticipation of analyzing both fixed and random effects models, we actually generated three counts for each covariate pattern. For the fixed effects model these counts were independent but for the random effects model they were correlated. For each simulation configuration we generated 1000 data sets with 32 observations each. The regression parameters were kept the same throughout the simulations and are provided in the equations

(14) $$\log\left(\lambda_{i}\right)\;\;=\;\;-2-0.3X_{1}+0.2Z_{1}+0.5Z_{2}$$

and

(15) $$\log\left(\mu_{i}\right)\;\;=\;\;-1+0.5X_{1}-0.5Z_{1}+0.1Z_{2}$$

We considered various values of the sensitivity and specificity and random effect variance, $\sigma^{2}$.

Previously, Edwards *et al.* [13] and Stamey *et al.* [9] performed simulations for the fixed effects model for the situation of the correctly known misclassification and prior information in the form of validation data that is centered on the true value. We repeated simulations similar to theirs and verified the fixed effects models perform very well in terms of bias and coverage when either the correct values for se and sp are used (Model 1) and when the priors are centered at the true values (Model 2). That is, when correctly modeling the misclassification, there is very little bias and coverage of the 95% intervals is close to nominal. We next consider this same situation for the random effects model. Specifically, we generated three correlated counts for each covariate pattern for values of the random effect variance of 0.1, 0.25, 0.5 and 0.75. We did this for se=0.9 and sp=0.8 and for se=0.9 and sp=0.6. We analyzed the data both with the naive model where the misclassification is ignored and the model where the misclassification is accounted for with priors for se of $\text{Beta}\left(45,5\right)$ and sp of $\text{Beta}\left(40,10\right)$ for both cases of misclassification parameters. These priors have means of 0.9 and 0.8 with standard deviations of 0.042 and 0.056, respectively. We focus on the results for $\beta_{1}$ and $\gamma_{1}$ but the general patterns are similar for all parameters. In Table 1 we display the averages of the posterior means for both the naive and “corrected” models for the se=0.9 and sp=0.8 and the se=0.9 and sp=0.8 cases across the values for $\sigma^{2}$. Table 2 and Table 3 report, for the same scenarios, 95% interval widths and coverages, respectively. In both cases for all parameters, the naive model yields biased estimators with empirical coverage probability below nominal, while the corrected model has posterior means close to the truth and coverage close to 95%. It is also interesting that the naive model has narrower intervals. Accounting for the misclassification increases the uncertainty in the model leading to wider intervals. The narrower intervals for the naive case contribute to the below nominal coverage as it leads to estimates being “precisely wrong”. That is, biased and overly confident.

**Table 1:** Average posterior means across 1000 simulations (truth: $\beta_{1}=-0.3$, $\gamma_{1}=0.5$).

se=0.9, sp=0.8	$\sigma^{2}$	$\beta_{1}$ (Naive)	$\beta_{1}$ (Model 4)	$\gamma_{1}$ (Naive)	$\gamma_{1}$ (Model 4)
	0.10	–0.11	–0.30	0.43	0.51
	0.25	–0.11	–0.32	0.42	0.50
	0.50	–0.10	–0.33	0.40	0.50
	0.75	–0.08	–0.34	0.39	0.50
se=0.9, sp=0.6	$\sigma^{2}$	$\beta_{1}$ **(Naive)**	$\beta_{1}$ **(Model 4)**	$\gamma_{1}$ **(Naive)**	$\gamma_{1}$ **(Model 4)**
	0.10	–0.07	–0.31	0.36	0.50
	0.25	–0.06	–0.32	0.35	0.50
	0.50	–0.05	–0.33	0.34	0.49
	0.75	–0.05	–0.33	0.32	0.49

**Table 2:** Average width of 95% intervals across 1000 simulations.

se=0.9, sp=0.8	$\sigma^{2}$	$\beta_{1}$ (Naive)	$\beta_{1}$ (Model 4)	$\gamma_{1}$ (Naive)	$\gamma_{1}$ (Model 4)
	0.10	0.52	0.66	0.46	0.53
	0.25	0.70	0.88	0.66	0.80
	0.50	0.93	1.15	0.89	1.06
	0.75	1.09	1.37	1.02	1.28
se=0.9, sp=0.6	$\sigma^{2}$	$\beta_{1}$ **(Naive)**	$\beta_{1}$ **(Model 4)**	$\gamma_{1}$ **(Naive)**	$\gamma_{1}$ **(Model 4)**
	0.10	0.53	0.76	0.44	0.56
	0.25	0.70	0.95	0.63	0.78
	0.50	0.91	1.21	0.86	1.08
	0.75	1.07	1.44	1.02	1.31

**Table 3:** Average coverage of the 95% intervals across 1000 simulations.

se=0.9, sp=0.8	$\sigma^{2}$	$\beta_{1}$ (Naive)	$\beta_{1}$ (Model 4)	$\gamma_{1}$ (Naive)	$\gamma_{1}$ (Model 4)
	0.10	0.72	0.95	0.90	0.96
	0.25	0.81	0.95	0.91	0.94
	0.50	0.88	0.96	0.91	0.95
	0.75	0.90	0.96	0.93	0.95
se=0.9, sp=0.6	$\sigma^{2}$	$\beta_{1}$ **(Naive)**	$\beta_{1}$ **(Model 4)**	$\gamma_{1}$ **(Naive)**	$\gamma_{1}$ **(Model 4)**
	0.10	0.59	0.96	0.74	0.95
	0.25	0.74	0.94	0.86	0.95
	0.50	0.81	0.96	0.86	0.94
	0.75	0.85	0.96	0.87	0.94

### 5.1. Robustness Considerations

**Figure 6:**
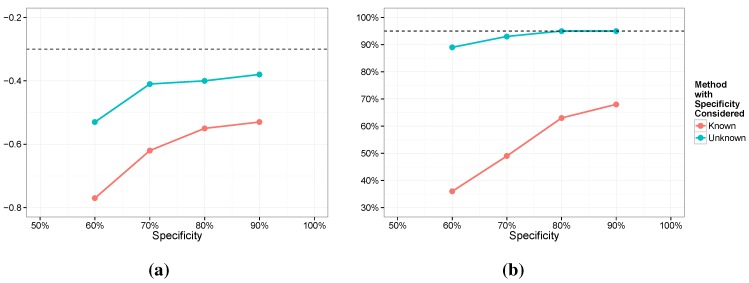
Posterior means (**a**) and coverage rates (**b**) for *γ*; se=0.75.

**Figure 7:**
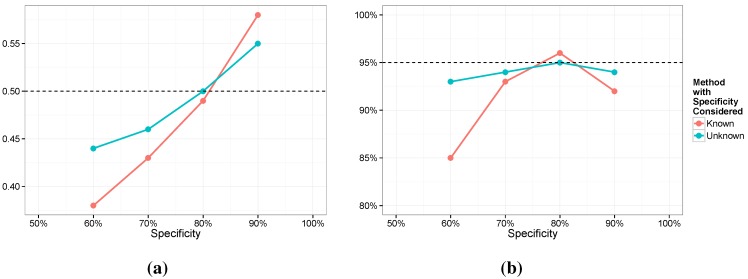
Posterior means (**a**) and coverage rates (**b**) for *β*; se=0.75.

We next investigated robustness. Specifically, we are interested in the impact of imperfect estimation of sensitivity and specificity. We focus on Models 3 and 4 here but the results were similar for Models 1 and 2. For Model 3 we assumed a value of 0.7 for se and 0.8 for sp. For Model 4 we centered the priors on these same values with a Beta $\left(35,15\right)$ for se and a Beta $\left(40,10\right)$ for sp (with means of 0.7 and 0.8 and standard deviations of 0.064 and 0.056, respectively). For the simulation we fixed se at 0.75, shifted mildly from the true value. For the specificity, we considered a range of values, sp=0.9,0.8,0.7,0.6. In Figure 6 and Figure 7 we provide the average posterior means along with the coverage of the nominal 95% intervals for both $\beta_{1}$ and $\gamma_{1}$. The most notable feature of the results is that while the posterior means are biased for both models, Model 4 is not nearly as biased and holds the coverage close to nominal. Conversely, Model 3 seems to be surprisingly sensitive. It is interesting to note that if we generate the data from the exact distribution assumed, that is, a value of 0.7 for se and 0.8 for sp, then estimation in Model 3 exhibits little bias and has 95% coverage. However, we see that estimation of $\gamma_{1}$ is quite poor in every case and the coverage for $\beta_{1}$ dips for lower values of the specificity.

## 6. Conclusions

In this paper we have extended previous work on count regression models with misclassification by including a random effect to allow for overdispersion commonly encountered in observational data. Using graphical models we have attempted to make the assumptions and form of the models more accessible to a general audience. Simulation results confirmed the performance of the model demonstrating the improvement over the naive model that ignores the misclassification can be substantial. We hope this work motivates researchers not only to account for misclassification but to consider the wide range of non-sampling bias that can be found in their observational data and to apply appropriate tools to fully address the problems that can arise. Future work includes the development of software for epidemiologists and public health researchers to address misclassification in Poisson and related count models used for public health data.
